# Oils from Transgenic Flax Lines as Potential Chemopreventive Agents in Colorectal Cancer

**DOI:** 10.3390/biomedicines11092592

**Published:** 2023-09-21

**Authors:** Tomasz Gębarowski, Benita Wiatrak, Izabela Jęśkowiak-Kossakowska, Magdalena Grajzer, Anna Prescha

**Affiliations:** 1 Department of Biostructure and Animal Physiology, The Wroclaw University of Environmental and Life Sciences, Kożuchowska 1/3, 51-631 Wroclaw, Poland; 2 Department of Pharmacology, Faculty of Medicine, Wroclaw Medical University, Mikulicza-Radeckiego 2, 50-345 Wroclaw, Poland; benita.wiatrak@umw.edu.pl; 3 Department of Basic Medical Sciences, Wroclaw Medical University, Borowska 211, 50-556 Wroclaw, Poland; 4 Department of Dietetics and Bromatology, Wroclaw Medical University, Borowska 211, 50-556 Wroclaw, Poland; magdalena.grajzer@umw.edu.pl (M.G.); anna.prescha@umw.edu.pl (A.P.)

**Keywords:** chemoprevention, seed oils, flaxseed

## Abstract

Colorectal cancer is a major global health concern, and the need for effective chemopreventive agents is paramount. This study aimed to evaluate the potential of oils from transgenically modified flax for the prevention of colorectal cancer, in relation to the oil concertation. Flaxseed oils were obtained from traditional (Nike) and genetically modified flax lines (M and B). Cell viability assays were performed on various cancer cell lines, including colon adenocarcinoma cells. Flaxseed oil B exhibited the strongest anti-proliferative properties compared to the reference drugs and other oils. Additionally, M and B oils showed enhanced accumulation of Rhodamine 123 and increased apoptosis in colorectal cancer cells. M oil exhibited the highest levels of p53 protein. Notably, the tested transgenic oils did not induce metastasis and displayed stronger inhibition of COX-1 compared to COX-2. These data indicate the utility of flaxseed oils, especially from the M line, as adjuvants in colorectal cancer treatment, targeting the colon specifically.

## 1. Introduction

Cancer stands second only to cardiovascular diseases as a leading cause of global mortality. Characterized by uncontrolled cell division due to genetic mutations affecting cell cycle proteins, cancer encapsulates a vast spectrum of over 100 diseases. By 2030, the global cancer burden is predicted to amplify, with new cases and deaths estimated at 22 million and 13 million, respectively [1,2].

Colorectal cancer (CRC), ranking third in men and second in women, demonstrates a concerning upward trend, especially in Western nations where dietary habits lean towards higher consumption of red and processed meats [3]. Diet, complemented by physical activity and body weight, is pivotal not only in the genesis of cancer but also in determining patient prognosis and quality of life [4,5].

In the realm of cancer prevention and management, diet plays an indispensable role. The American Cancer Society underscores the virtues of a diet replete with whole grains and plant-derived nutrients. Foods rich in phenolic compounds, like olive oil, are particularly advocated, given their antioxidant and anti-inflammatory prowess [4,6].

Chemoprevention, defined as the use of natural or synthetic agents to thwart or delay carcinogenesis, has gained significant traction over the years. The multifaceted mechanisms employed by chemopreventive compounds range from the modulation of apoptosis to the regulation of cell cycles through kinases [5]. Cancer chemoprotection can be achieved through the enhanced detoxification of reactive free radicals, a reduction in the production of reactive nitrogen and oxygen, the induction of repair pathways, as well as a reduction in chronic inflammation. The chemopreventive factors in colorectal cancer include non-steroidal anti-inflammatory drugs, minerals, and vitamins [7,8,9].

A CRC prevention strategy may involve the use of dietary supplements providing higher concentrations of specific nutrients with anti-inflammatory and antioxidant properties, modulating the gut microbiota, maintaining intestinal homeostasis, and regulating immune responses [10]. In that sense, seed oils, especially cold-pressed seed oils, laden with polyunsaturated fatty acids (PUFA), sterols, tocopherols, carotenoids, and phenolic compounds, have been the focal point of numerous studies. The synergistic interplay of these components at varying concentrations can potentially bolster cancer therapeutics [10,11,12,13,14]. Plant oils are ideal candidates as chemopreventive agents because they are non-genotoxic and are able to effectively inhibit the formation of 17b-estradiol epoxide (E2), which is responsible for the induction of the basic mechanism of breast cancer initiation [15]. Oil extracted from *Pistacia lentiscus var. chia* suppressed tumor growth in experimental colon cancer models in mice [16]. In addition, pomegranate seed oil suppressed azoxymethane-induced colon carcinogenesis in rats and increased the expression of PPARγ protein in the colon mucosa [17]. *Ocimum sanctum* seed oil showed chemopreventive activity, which was confirmed in a mouse model [18]. Furthermore, juniper berry oil (JB) acts as a chemopreventive dietary agent, inhibiting cell proliferation and COX-2 expression and inducing apoptosis, resulting in a significant reduction in colon tumor formation [19].

As challenges like drug resistance encroach upon the efficacy of traditional chemotherapy, the scientific community is gravitating towards natural, cost-efficient agents that counteract CRC [20,21,22,23]. Seeds oils, including rose and raspberry seeds oils, have demonstrated chemopreventive effects in colon cancer cell models and hold promise as prophylactic agents or supplements to traditional chemotherapy [24,25].

In this backdrop, transgenic seeds, fashioned through genetic modifications to imbibe specific, new, or enhanced traits, have garnered considerable attention in modern agriculture as products with potential benefits and implications [26]. In the case of flaxseed oil, transgenic seeds offer several potential advantages, including an enhanced nutritional profile. For example, they can be modified to contain higher levels of beneficial components, such as α-linolenic acid (ALA), sterols, tocopherols and lignans, which have been associated with various health benefits, including cardiovascular health and anti-inflammatory properties. By increasing the concentration of these desirable components, these oils may offer enhanced nutritional and chemopreventive value [27,28,29].

Within this study’s ambit, we intend to unravel the implications of cold-pressed oil from the seeds of genetically modified flax lines, on doxorubicin-sensitive and resistant CRC cells. By delving into the compositional variances of these oils, our objective is to elucidate findings that could potentially inform and improve colorectal cancer (CRC) preventive and therapeutic strategies.

## 2. Materials and Methods

### 2.1. Material

The material for the study consisted of flaxseed oil cold-pressed in a hydraulic screw-press at room temperature from the flaxseed variety Nike and 2 transgenic lines, M and B [28].

Transgenic line B was obtained by cloning the β-1,3-glucanase gene from *Solanum tuberosum* into the genome of plants of the Nike variety. The aim of this modification was to enhance the plants’ resistance to fungal pathogens. The results included a moderate increase in resistance to *Fusarium*, a rise in the number of seeds produced by the plant, and a reduction in the total phenolic acid content in the seeds and green tissue. Conversely, a significant increase in the content of polyamines—putrescine and spermidine—were observed [26,29,30].

The M oil was obtained from the seeds of a Nike-derived transgenic genetically modified to produce polyhydroxybutyrate (PHB). Three genes encoding enzymes for the PHB synthesis pathway were derived from *Ralstonia eutropha* and introduced into the genome of the flax plants. In the tissues of the M line plants, there was not only a significant accumulation of PHB as expected, but also an increase in the levels of glucose and starch [27,29].

Considering the distinct active ingredient compositions of oils M and B, a 1:1(*v*:*v*) mixture was formulated to evaluate the potential synergistic effects of the combined components.

For the purpose of in vitro studies, the flaxseed oil emulsions were prepared following the protocol established by Skorkowska-Telichowska et al. [31]. Briefly, one gram of soybean lecithin was emulsified with 0.5 mL of Tween 80, 2.5 mL of oil and 5 mL of an aqueous phase containing glycerol 25% *v*/*v*. Oils were reported to the medium in the form of emulsions. The emulsions prepared for addition to the culture contained 5% of the tested oils. The emulsions of oils were added to the culture medium at concentrations of 0.2%, 0.5%, 1%, 2%, and 5%. Oil-free emulsions at 5% were added as controls. The emulsions, which were added to the culture, were stable and did not delaminate. Flaxseed oils were tested at final concentrations of 0.1 mg, 0.25 mg, 0.5 mg, 1.0 mg, and 2.5 mg per 1 mL of the medium at room temperature and in sterile conditions. The range of concentrations of the tested oils used is not toxic to cells and allows for chemopreventive effects in cancer cell model [25]. The MB oil emulsions were prepared by mixing 1:1 B and M oil (*v*/*v*).

### 2.2. Oils’ Chemical Characteristics

#### 2.2.1. Fatty Acids Composition

The composition of fatty acids in the tested oils was determined by gas chromatography on gas chromatograph 6890 N (Agilent Technologies, Santa Clara, CA, USA); a capillary column SP-2560 100 m × 0.25 mm × 0.2 μm (Supelco, Sigma Aldrich, St. Louis, MO, USA) was used for the separation of volatile fatty acid methyl esters. The retention times of the individual peaks obtained on the chromatograph of the test sample were related to the retention times of the methyl ester mixture of standard substances, which were fatty acids with chain lengths from C4 to C24 (37 compounds). The determination was performed according to the modified method of Prescha et al. [32].

#### 2.2.2. Phytosterols Composition and Content

Phytosterol composition contents were determined by gas chromatography using a capillary column to separate silylated phytosterol derivatives. Oil samples were prepared for determination according to the modified method of Shukla et al. [33]. To 40 μL of the oil sample, 40 μL of a 1 mg/mL chloroform 5α-cholestane solution was added as an internal standard. Then, after 2 mL of 2 M KOH in anhydrous methanol was added to the sample, the mixture was heated for 45 min at 60 °C, with shaking every 10 min or so. After the sample had cooled to room temperature under a running stream of cold water, 1 mL of distilled water, 2 mL of n-hexane, and 0.5 mL of absolute ethanol were added in sequence. The whole mixture was vigorously shaken for 5 min using a horizontal shaker, followed by centrifugation for 6 min at 2500 rpm. The upper hexane layer, containing the unsaponifiable fat fraction, was transferred with a Pasteur pipette into a glass tube. The extraction of the unsaponifiable fraction was repeated with an additional 2 mL of n-hexane. The combined extracts were then evaporated using a stream of gaseous nitrogen. After the solvent was removed, 150 μL of the silylating reagent and 150 μL of pyridine were added to the sample, which was then incubated for 45 min at 60 °C. Silyl sterol derivatives were separated on a non-polar HP-1 100% Dimethyl Siloxane capillary column (30 m × 250 μm × 0.25 μm). The sample injection per column was 2 μL. Hydrogen was used as the carrier gas with a pressure of 21.9 psi and a flow rate of 40 mL/min. Air was used as the make-up gas with a flow rate of 450 mL/min, and nitrogen was used as the masking gas with a flow rate of 45.0 mL/min. The detector temperature was 310 °C. Chromatographic separation was carried out on a Clarus^®^ SQ 8 gas chromatograph/mass spectrometer (GC/MS)(PerkinElmer, Waltham, MA, USA) in a temperature program with an initial temperature of 250 °C for 5 min, followed by an increment of 5 °C/min to a final temperature of 290 °C, which was maintained for 13.5 min.

#### 2.2.3. Composition of Tocopherols and 8-Plastochromanol Content along with Carotenoids Composition and Content

First, the unsaponifiable fraction, containing the tocochromanols and carotenoids, was extracted from the oils. Saponification was carried out at room temperature in the presence of ascorbic acid, which was added to the sample before saponification to prevent the loss of oxidation-sensitive tocopherols and carotenoids. Saponification and extraction of the compounds were carried out according to the method previously described by Fromm et al. [34].

Second, the identification and quantification of analytes were carried out on an ACQUITY UPLC system (Waters Corporation, Milford, CT, USA). Analytes were separated on a 100 mm ×  2.1 mm, 1.7 μm Acquity UPLC CSH C18 column using the following solvent gradient mixtures: mixture A: 30% methanol, 50% acetonitrile, 20% water (*v*/*v*); mixture B: 50% methanol, 50% acetonitrile (*v*/*v*). The flow rate of the mobile phase was 0.5 mL/min. Chromatograms were recorded at the following wavelengths: γ-and δ-tocopherol and plastochromanol-8 at 298 nm, α-tocopherol homolog at 293 nm; and compounds from the carotenoid group at 445 nm. The identifications and the quantifications were based on an external standard and an external standard calibration curve [35].

#### 2.2.4. Polyphenolic Compounds and Composition

The determination of phenolic compounds from the oils was preceded by the isolation of phenolic compounds from the oils by solid-phase extraction (SPE) based on the method described previously by Pirisi et al. [36], followed by identification and quantitative analysis of these compounds by ultra-pressure liquid chromatographic analysis. Analysis was performed using a Waters Acquity UPLC system (Waters Corporation, Milford, CT, USA) connected to a PDA detector 200–500 nm and mass spectrometer Xevo-Q-TOF (Waters, Milford, CT, USA). Separation was performed, as in the case of tocochromanol and carotenoid determinations, on a C18 column with the same parameters. The injection volume was 5 µL. Solvent mixtures A and B were used for separation. Mixture A contained 99.5% water and 0.5% formic acid (*v*/*v*). Mixture B was 99.5% acetonitrile and 0.5% formic acid (*v*/*v*). The flow rate of the mobile phase was 0.6 mL/min. Chromatograms were recorded at wavelengths of 280 and 320 nm [35].

### 2.3. In Vitro Studies

#### 2.3.1. Cell Line and Conditions

The cytotoxicity was evaluated using five cancer cell lines: lung cancer cells (A549), breast cancer cells (MCF7), blood cancer cells (CCRF/CEM) and two colorectal carcinoma cell lines—LoVo and LoVo/DX (doxorubicin-resistant)—which was prepared by incubation of LoVo with a low concentration of doxorubicin for three months. All cancer cell lines except LoVo/DX were obtained from ATCC (Manassas, VA, USA). The cytotoxicity assessment was also performed for the colon epithelium (CCD 841 CoTr), which was obtained from ATCC. All cells were cultured in the recommended medium at 5% CO_2_, 37 °C, 95% humidity. An EMEM medium was used for the A549 and MCF 7 cells, RPMI 1640 for CCRF/CEM, DMEM F12 for colorectal cancer, and DMEM without phenol red for CCD 841 CoTr. All media were supplemented with 10% FBS, 2 mM L-glutamine, and 25 μg/mL gentamicin. The cell culture reagents used in the study were purchased from Biological Industries (Beit-Haemek, Israel) and the cell culture plastics bridged in the study were purchased from SPL Life Sciences (Pochon, Republic of Korea). The study used an incubator ILC 180 SMART PRO from POL-EKO (Wodzisław Śląski, Poland).

#### 2.3.2. Viability Assay

The MTT assay was performed to allow for an estimation of the viability of cells. After 24 h of incubation with oils in the form of an emulsion or Doxorubicin (Sigma Aldrich, St. Louis, MO, USA), the supernatant was removed, the cells were rinsed with PBS, then tetrazole salt at a concentration of 1 mg/mL was added into each well. In the next 2 h, the culture was incubated in a CO_2_-incubator, then crystals of formazan were dissolved in isopropanol by shaking for 30 min. After that, the spectrophotometric measurement was taken at 570 nm using a Multiskan GO microplate reader Thermo Fisher Scientific (Waltham, MA, USA).

#### 2.3.3. Accumulation of Rhodamine 123

**Figure 1:**
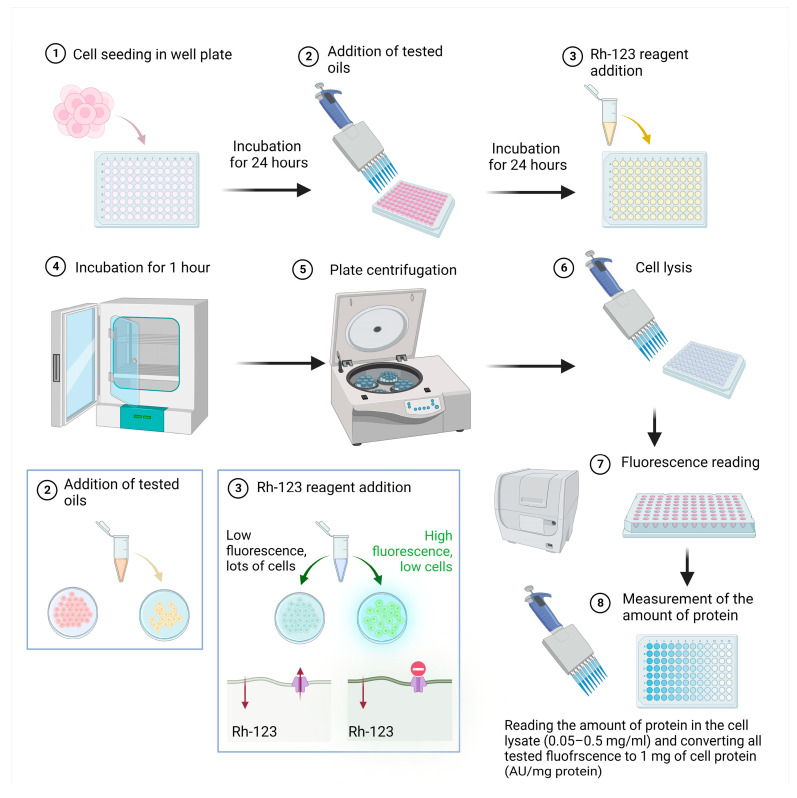
Flowchart of the assessed effect on P-glycoprotein (P-gp) transport function by the tested oils in cells of the LoVo and LoVo/DX lines using the using a VICTOR2 microplate reader. Created with BioRender.com, accessed on 20 August 2023.

To assess the effect on P-glycoprotein (P-gp) transport function, we performed a rhodamine 123 (Rh-123) accumulation assay on the cells. A solution of Rh-123 (Sigma, St. Louis, MO, USA) at a concentration of 10 mM in a 1:1 DMSO: water mixture was used for the experiment. Cells were seeded into sterile 96-well opaque-wall plates in a volume of 100 µL culture medium of 2 × 104 cells of the LoVo and LoVo/DX lines and cultured for 24 h. At the beginning of the experiment, solutions of the tested elicitors in a culture medium (without FBS) were added to the test wells at a volume of 100 µL/well. Cells were incubated with the test oils in the form of an emulsion for 18 h. After this time, Rh-123 was added to the wells to a final concentration of 12.5 µM and incubated for 60 min. After incubation, the plates were centrifuged for 10 min, 500× *g*, and the supernatant from the pellet was removed. The cell pellet was dissolved (150 µL/well) in 20 mM Tris-HCl (pH 7.7) containing 0.2% sodium dodecyl sulphate (SDS) for cell lysis and release of the intracellular fluorescent substrate. Fluorescence was measured using a VICTOR2 microplate reader (PerkinElmer, Waltham, MA, USA) ex. 485/em. 538 nm. From each test sample, 6 µL of solution was taken for total protein determination using the Bradford method. The need to measure and convert the results obtained into 1 mg of protein was due to the effect of the oils on cell proliferation. The scheme of the experiment is shown in Figure 1.

#### 2.3.4. Detection of Apoptosis

The test oils were added at concentrations of 0.5–2.5 mg per 1 mL for 24 h. The medium from each well was collected into pre-prepared centrifuge tubes, as there may have been non-adherent, dead, or apoptotic cells in the supernatant. The wells were then washed with a trypsin–EDTA solution, and the supernatants were also collected in tubes. Cells were re-treated with trypsin–EDTA for 2 min at 37 °C and collected into tubes. The tubes were then centrifuged at 600× *g* for 10 min at 20 °C for 10 min. The cell pellet was resuspended in 100 μL of HEPES-NaOH pH 7.5 buffer, a mixture of annexin V-FITC fluorochromes and propidium iodide (Sigma Aldrich, St. Louis, MO, USA) was added and left in the dark for 10 min. The preparations were analyzed using an Arthur^TM^ image cytometer (NanoEnTek Inc., Seoul, Republic of Korea). In addition, an assessment of apoptosis and necrosis in wells on culture plates was performed. After removing the medium, HEPES-NaOH pH 7.5 buffer was added to the well, a mixture of annexin V-FITC fluorochromes and propidium iodide and 10 µM Hoechst 33342 for 20 min in a CO_2_-incubator. Then, photographs were taken using an EVOS FL microscope Thermo Fisher Scientific (Waltham, MA, USA).

#### 2.3.5. Measurement of the Amount of p53 Protein

The measurement of p53 protein content in cell lysates was performed with the use of the pan p53 ELISA kit (Roche). According to the manufacturer’s instructions, the tested cell lysates and selective anti-p53 antibodies were added to the antibody-coated wells. During the test, platelet-bound and p53-bound antibodies were combined. After adding tetramethylbenzidine (TMB)—a substrate for horseradish peroxidase (antibody-conjugated)—to the wells, TMB absorbance was read by measuring absorbances in a spectrophotometer at 450 nm and the amount of p53 protein was calculated. In the cell lysates, the measurement of total protein was also performed by the Bradford method using the Protein Quantification Kit-Rapid (Fluka part of Sigma, St. Louis, MO, USA). The final test result was expressed in ng p53 per mg of total protein.

#### 2.3.6. Cell Cycle

Cells, after 24 h of incubation with the oils, were fixed, permeabilized, and incubated with a fluorescently labeled antibody (FITC) directed against BrdU in order to determine the amount of BrdU incorporated into the cells’ DNA. DNA was simultaneously stained with 7-aminoactinomycin D (7-AAD) binding to total DNA. The test was performed using a flow cytometer on an Arthur^TM^ image cytometer.

#### 2.3.7. Cell Migration

**Figure 2:**
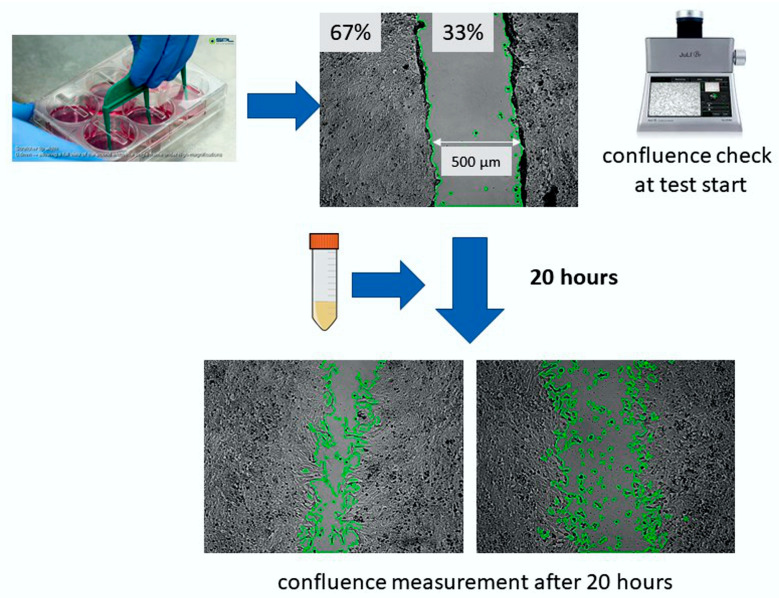
Flowchart of the effect on cell invasiveness of LoVo and LoVo/DX lines using the SPLScar™ Scratcher cell migration assay system adapted with permission from Ref. [37].

To assess the effect of the test compounds on tumor metastasis, a migration assay was performed. The cell culture monolayer was scratched using the SPLScar™ Scratcher cell migration assay system (Figure 2). Then, the tested oils were added to the cell cultures. In the migration assay, the lowest active concentration was selected for all tested oils. Photographs were taken with a Juli Br microscope (NanoEnTek Inc., Seoul, Republic of Korea), then the cell cultures were incubated for 24 h and the scratch was photographed again. In addition, the rate of crack overgrowth was analyzed by monitoring the culture using a Juli microscope. Using the ImageJ 1.54d open software platform, the length of the scratch was measured after its preparation, 24 h after incubation with the test compounds, and at 15 min intervals to determine the rate of overgrowth.

#### 2.3.8. Proliferation Effect of Ki67 Protein

Ki-67 was determined in cells of the colon cancer line LoVo treated with the tested flaxseed oils emulsions by flow cytometry. LoVo colorectal cancer cells were collected, counted, and pelleted according to standard procedures. While stirring, 5 mL of cold 70% ethanol was added dropwise to the cell pellet (1 − 5 × 10^7^ cells). They were then incubated at −20 °C for 2 h minimum, and 30–40 mL of the washing buffer (PBS with 1% FBS, 0.09% NaN_3_ pH 7.2) was added to the fixed cells. The cells were centrifuged for 10 min at 1000 rpm. The supernatant was aspirated and washed again with 30–40 mL of the washing buffer. It was centrifuged at 1000 rpm for 10 min and the supernatant was aspirated. Cells were resuspended to a concentration of 1 × 10^7^/mL (1 × 10^6^/100 µL). Then, 100 µL of cell suspension was transferred to each fresh tube, and 20 µL of appropriately diluted antibody was added, according to the protocol, to the above tubes. The tubes were incubated at room temperature for 20–30 min in the dark, then washed with 2 mL of the PBS washing buffer at 1000 rpm for 5 min. The supernatant was aspirated, and 0.5 mL of the PBS wash buffer was added to each tube. To the FITC-conjugated antibodies, 10 µL of the PI staining solution for PE-conjugated antibodies was added, 20 µL of BD Via-Probe™ Cell Viability Solution was added to each tube. Samples were analyzed with the CytoFLEX S Flow Cytometer, (Beckman Coulter, Indianapolis, IN, USA).

#### 2.3.9. In Vitro Cyclooxygenase Inhibition Assay

The activity of COX peroxidase was assessed using the colorimetric method. The reduction of PGG_2_ (prostaglandin G_2_) to PGH_2_ is due to the oxidation of N,N,N′,N′-tetramethyl-p-phenylenediamine (TMPD). The result is a color change measured at 590 nm (Variuscan Go microplate reader). The kit supplied by the manufacturer contains 150 μL of assay buffer, 10 μL of heme and 10 μL of COX-1 or COX-2. All compound samples were prepared in triplicate by adding 10 μM of test compounds and 10 μM methanol, ethanol and DMSO, respectively. Then, 20 μL of TMPD was added to all wells. Finally, arachidonic acid was added to activate the reaction, which lasted for 2 min. The oxidation of the TMPD was then assessed using the Multiskan GO microplate reader (Thermo Fisher Scientific (Waltham, MA, USA).

### 2.4. Statistical Analysis

The normal distribution was checked using the Shapiro–Wilk test, and the equality of variances was checked by Levene’s test. ANOVA and Tukey post hoc tests were performed. The level of statistical significance was assumed to be *p* < 0.05. The relationship between IC_50_ inhibitory concentration and polyphenol group compounds was evaluated using the correlation coefficient in Statistica 13.3 software.

## 3. Results

### 3.1. Oil’s Chemical Characteristics

#### 3.1.1. Fatty Acids

The oils from Nike, and its transgenic line M, contained about 44% ALA (C18:3 n-3), and more than 19% C18:2 n-6 (Table 1). Oil from the B line had the lowest amount, not exceeding 42%, of ALA among the studied oils. No differences were observed in the content of other fatty acids determined in the seed oils of Nike and its transgenic lines.

According to the records of the European pharmacopeia, the composition of linseed oil includes palmitic acid: from 3.0% to 8.0%; palmitoleic acid: not more than 1.0%; stearic acid: from 2.0% to 8.0%; oleic acid: from 11.0% to 35.0%; linoleic acid: from 11.0% to 24.0%, ALA: from 35.0% to 65.0%; arachidic acid: not more than 1.0%. In the range of parameters studied, the oils tested met the requirements of the document.

#### 3.1.2. Phytosterols Composition and Content

The total content of phytosterols in the analyzed flaxseed oils averaged 681.7 to 652.0 mg per 100 g of oil (Table 1). The highest number of total sterols, exceeding 680 mg/100 g, was found in Nike oil. It was observed that oils from the M and B lines contained significantly less (*p* < 0.001) phytosterol than the Nike control.

The main sterol compound of flaxseed oils was β-sitosterol. It accounted for an average of 44.5% of the phytosterol fraction. The M oil (296.6 mg/100 g), and its control Nike (291.9 mg/100 g), contained the most of this compound. In the B line, the content of this compound was significantly lower than that in the control and in M oil. The highest amounts of stigmasterol were determined in the oil obtained from B flax and Nike (42.4 mg, 40.2 mg, respectively) and were significantly higher than those found in the M oil line.

In all oils, the contents of tocopherols and plastochromanol-8 were similar (Table 2). The richest source of tocopherols was oil from the Nike and line M, containing ca. 352 mg/kg, and in line B, total tocopherols significantly differed from that in the Nike oil (Table 2). γ-Tocopherol accounted for an average of 94%, while α and δ forms accounted for about 3.5 and 3.3% of total tocopherols in the B and M oil lines.

In addition to tocopherols, plastochromanol-8, another antioxidant belonging to the tocochromanol group, was found in flaxseed oils. The lowest content of this compound was found in M oil (80.5 mg/kg), while the highest content was found in Nike 107.71 mg/kg.

The tested oils differed in the content of both total and individual carotenoids (Table 2). Their highest concentration was determined in the unmodified Nike variety (25.0 mg/kg). The sum of lutein and zeaxanthin accounted for the largest share of the total carotenoid content of the tested oils (up to about 90%), which had the greatest impact on the differences in carotenoid levels in these oils. Oils from the Nike line and its transgenic line B had the highest concentration of β-carotene (4.5 and 4.1 mg/kg, respectively) In contrast, the content of this compound in the transgenic M line oil was 2.05 mg/kg. β-Cryptoxanthin in the unsaponifiable fraction of the tested flaxseed oils was found in small amounts (ca. 0.2 mg/kg).

The phenolic acids vanillic, p-coumaric, o-coumaric and ferulic acids, as well as the aldehydophenols vanillin, syringic aldehyde, and coniferyl aldehyde, were identified in the tested oils (Table 2). Secoisolariciresinol from the lignan group was also found. The highest concentrations of phenolic compounds in these oils were determined in oil from transgenic line M, at 309 mg/kg, which was significantly higher than that established for both the Nike and B oils. Vanillic acid and vanillin were predominant in M oil and their concentrations were significantly higher than in those in the remaining studied flaxseed oils. In Nike oil, the phenolic compounds present in the highest amounts were vanillin and ferulic acid.

### 3.2. In Vitro Studies

#### 3.2.1. Viability Assay

The effect of the tested oils on the cellular vitality of cancer lines in the MTT reduction assay was evaluated. The human normal colon epithelial cells, CCD 841 CoTr, human colon adenocarcinoma cells (LoVo), human colon adenocarcinoma cells resistant to Doxorubicin (LoVo/Dx), human Caucasian lung carcinoma (A549), human breast cancer cell line with estrogen, progesterone and glucocorticoid receptors (MCF7), and Caucasian acute lymphoblastic leukemia A T lymphoblastoid line (CCRF/CEM) were used in the research. Table 3 presents the IC_50_ values, in which the vitality of the cultures was reduced by 50%. The antiproliferative activity against the mentioned cell lines of four linseed oils, NIKE, and transgenic varieties M, B, and MB was tested in comparison with the reference drug doxorubicin. The biological effects presented in Table 3 indicate that the tested oils exhibit chemopreventive properties. The strongest anti-proliferative properties were shown in the B oil for all cell lines in comparison with the reference drug and other oils. The strongest anti-proliferative properties of all the tested oils and the reference drug occurred against Caucasian acute lymphoblastic leukemia A T lymphoblastoid line (CCRF/CEM), human breast cancer cell line with estrogen, progesterone, and glucocorticoid receptors (MCF7), and human colon adenocarcinoma cells (LoVo). The values of IC_50_ were estimated based on nonlinear regression using the dependence of the biological effects on the molar concentrations of the compounds (four-parameter logistic model with Hill slope).

For the IC_50_ results obtained, correlation coefficients were calculated in relation to the polyphenol content of flaxseed oil. When evaluating the IC_50_ parameter, we expected the lowest possible values. A decrease in IC_50_ values correlates with an increase in polyphenol content. In the case of the LoVo line, significant negative correlations were obtained for the majority of comparable polyphenols. A similar effect was ringed for the MCF7 line. In the case of the LoVoDx line, the highest correlation coefficient was for Ferulic acid. The correlation coefficient was not determined for line A549 due to the lack of activity in some oils (Table 4).

#### 3.2.2. Accumulation of Rhodamine 123

**Figure 3:**
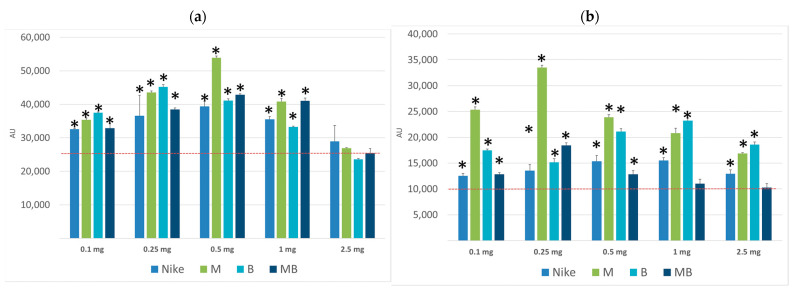
Effect of the tested oils on Rh-123 accumulation in cells of the LoVo (**a**); and LoVo/DX (**b**) lines (expressed in conventional units per mg protein; incubation time of cells with the tested oils—24 h. Control (LoVo) 26,582 ± 790; (LoVo/DX) 10,582 ± 512. The result is presented in fluorescence units read by a microplate reader converted to 1 mg of protein obtained by cell lysis (AU/mg). Results are averages of 5 independent experiments. Statistical significance of differences between the results for the test oils compared to the control (* *p* < 0.05).

P-glycoprotein activity was tested using the Rh-123 accumulation test. Accumulation of Rh-123 was observed for all tested oils, regardless of the concentration used, in the entire range of concentrations; however, the highest accumulation of rhodamine was observed after incubation with oils of LoVo colorectal cancer cells compared to the LoVo/Dx line. The highest rhodamine accumulation was achieved for the the LoVo cell line incubated with the emulsion containing 0.5 mg per 1 mL M oil, and in case of LoVo/Dx line the highest Rh-123 accumulation was obtained for 0.1 and 0.25 mg per 1 mL of M oil. The incubation with the 0.1 and 0.25 mg per 1 mL of B oil resulted in the higher Rh-123 accumulation in LoVo cell line in comparison with other oils. The opposite results were obtained for the doxorubicin-resistant colon cancer line LoVo/Dx incubated with B oil where a higher rhodamine accumulation then in other oils occurred in the 1.0 and 2.5 mg oil emulsion (Figure 3).

#### 3.2.3. Detection of Apoptosis

**Figure 4:**
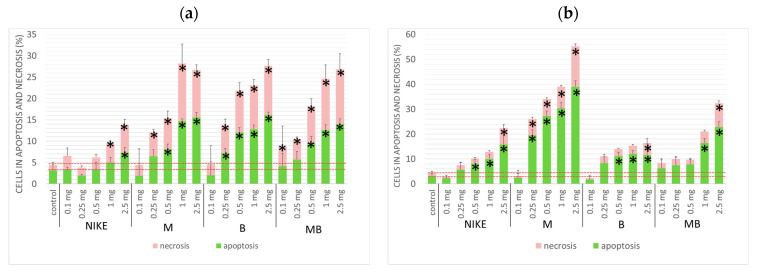
Apoptosis and necrosis of LoVo (**a**); and LoVo/DX (**b**) cells after 24 h incubation with the test oils at 5 concentrations (mg/mL). Results are presented as a percentage of apoptotic and necrotic cells. Results are averages of 5 independent experiments. Statistical significance of differences between the results for the test oils compared to the control (* *p* < 0.05).

Apoptosis is a process of programmed cell death and at the same time an important property of substances with anticancer activity. In turn, necrosis leads to uncontrolled cell death along with an inflammatory response in the surrounding tissues, which is considered a side effect of anti-cancer drugs. An increase of 50% in the frequency of apoptosis after 24 h of incubation was determined for the four tested oils, i.e., NIKE, M, B and MB, against the colon cancer line LoVo and LoVo/Dx. An increase in the frequency of apoptosis compared to necrosis occurred in the highest percentage of all oils against cells of the colon cancer line LoVo/Dx. For the LoVo colorectal cancer line, the percentage of apoptosis and necrosis appeared comparable for each of the oil concentrations tested. The highest percentage of apoptosis occurred for oils from transgenic lines M and B against the colon cancer line LoVo (Figure 4a). In the case of the colon cancer line resistant to doxorubicin, LoVo/Dx, the highest percentage of apoptosis was observed in the M oil (Figure 4b).

**Figure 5:**
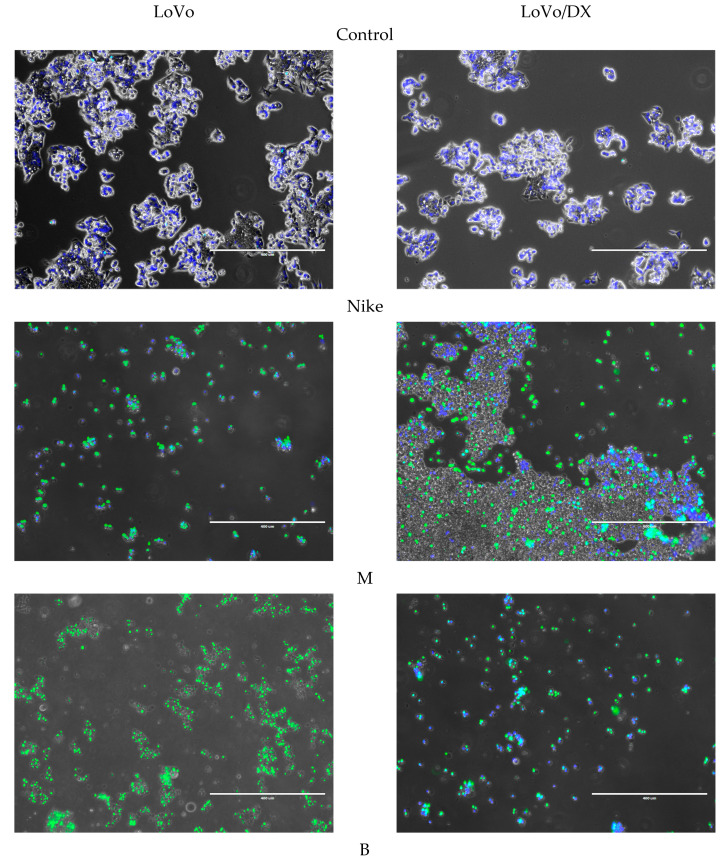

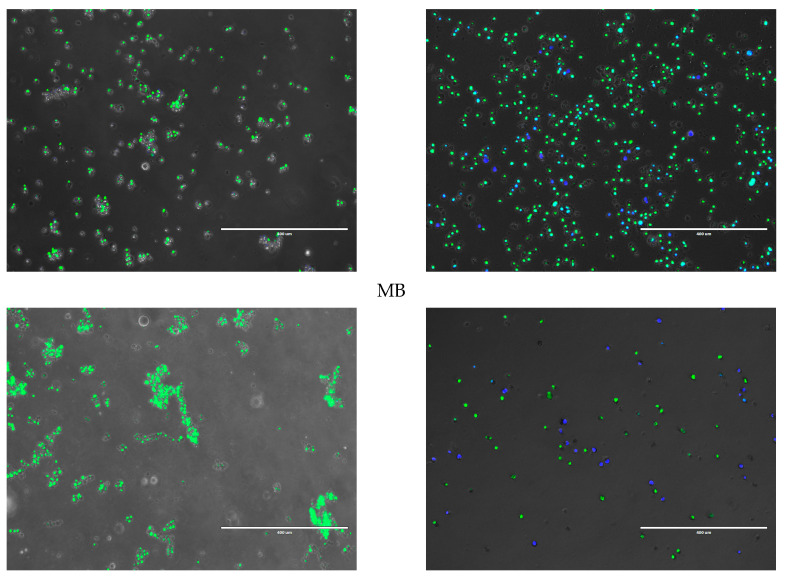
The obtained results confirmed the pro-apoptotic effect of all the tested oils in LoVo colorectal cancer and LoVo/Dx doxorubicin-resistant colorectal cancer cell cultures.

The obtained results confirmed the pro-apoptotic effect of all the tested oils in LoVo colorectal cancer and LoVo/Dx doxorubicin-resistant colorectal cancer cell cultures. After 24 h of incubation, the greatest pro-apoptotic effect was presented by two oils from lines M and B, both for the colon cancer lines LoVo and LoVo/Dx (Figure 5).

#### 3.2.4. Measurement of the Amount of p53 Protein

**Figure 6:**
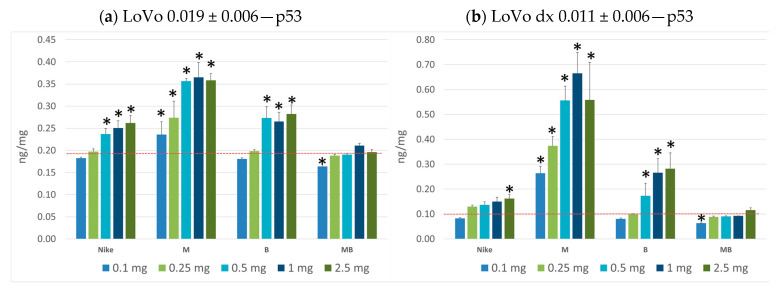
Results of p53 protein measurement using an enzyme immunoassay (ELISA) kit for studied oils in the entire selected range of concentrations for the LoVo (**a**); and LoVo/Dx (**b**) colorectal cancer lines. (* *p* < 0.05).

The p53 protein, which is a marker of apoptosis, is involved in both external (i.e., receptor) and internal (i.e., mitochondrial) apoptosis pathways [32], and was selected for an assay in colorectal cancer cells after treatment with the tested oils. The concentrations of p53 were analyzed in precise quantitative measurements using an enzyme immunoassay (ELISA) kit for all four flaxseed oils in the entire selected range of concentrations for the LoVo and LoVo/Dx colorectal cancer lines. In all oils in the studies, there was a proportional increase in the concentration of p53 protein with increasing conventional units per mg of protein on each tested cancer line. In general, higher amounts of p53 protein were found when testing oils on the LoVo colon cancer line compared to the LoVo/Dx colon cancer line (Figure 6a,b). For both the LoVo and LoVo/DX colorectal cancer lines, the highest p53 protein values were found in the M oil. This flaxseed oil induced programmed cancer cell death to the greatest extent.

#### 3.2.5. Cell Cycle

**Figure 7:**
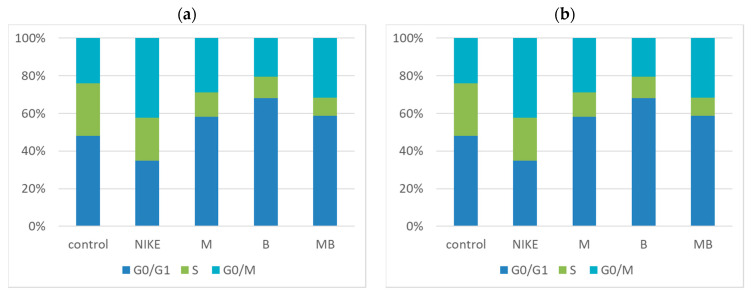
Cell cycle in LoVo (**a**); and LoVo/Dx (**b**) cells after 24 h incubation with test flaxseed oils at a concentration of 2.5 mg/mL. Results are presented as a percentage of cells in each phase. Results are averages of 5 independent experiments.

Cell cycle analysis was performed to see if the LoVo and LoVo/Dx tumor cells incubated with the test oils increased the number of cells in the proliferative phase (S phase) after 24 h of incubation. For all oils from the transgenic lines, fewer S-phase cells were found compared to those found in the NIKE oil, while an increase in the number of cells in the G0/G1 phase (Figure 7) was observed. Oils from the transgenic lines had a greater number of cells in the G0/G1 phase compared to the traditional NIKE line. The largest number of cells in the G0/G1 phase was found in the B oil against each of the tested cell lines. The obtained results indicate the beneficial effect of oils on colon cancer lines, as they direct cells to the programmed death of damaged cells at the first checkpoints of the cell cycle, especially in the G0 and G1 phases, which prevents the formation of two defective daughter cells in the next phase, M.

#### 3.2.6. Cell Migration

**Figure 8:**
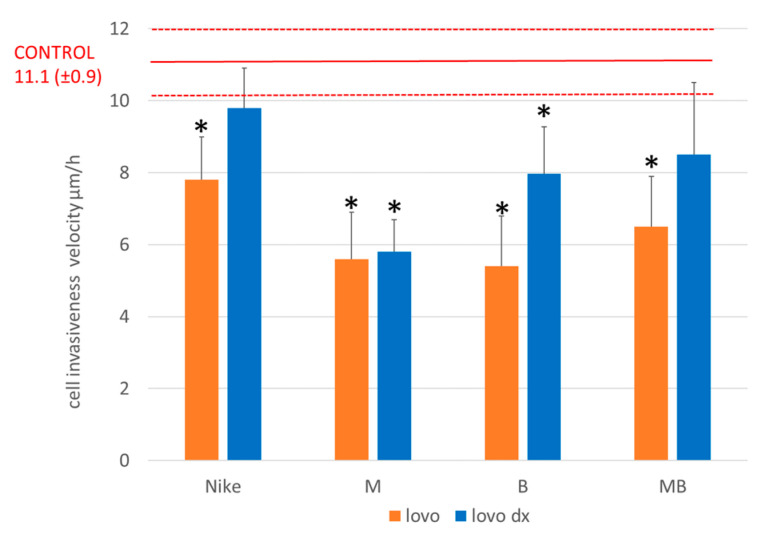
Effect on the invasiveness of LoVo and LoVo DX lineage cells measured by the average migration velocity over 20 h. A concentration of 0.25 mg/mL of test oil was used in the study (* *p* < 0.05).

Cell migration was performed to evaluate flaxseed oils as potentially therapeutic in inhibiting the formation of tumor metastasis. Cell migration was tested for all oils for both LoVo and LoVo/Dx colorectal cancer lines. The seeded LoVo and LoVo/Dx tumor cells were incubated until they formed a monolayer over the entire surface of the well. Then, a scratch test was performed by making a scratch in the monolayer and measuring its width. A concentration of 0.25 mg/mL of the tested oils was added to the LoVo and LoVo/Dx cells. Culture plates were incubated for 20 h. The crack width was then re-measured using the ImageJ open-source platform. Inhibition of LoVo and LoVo/Dx cell migration occurred for all tested oils, but greater inhibition of cell migration was observed for the LoVo line. The smallest cell growth in the LoVo culture occurred for oils from lines M and B, and for LoVo/Dx, from M oil (Figure 8). The width of the crack after 20 h, when no flaxseed oil was applied to the cells, was used as a control.

**Figure 9:**
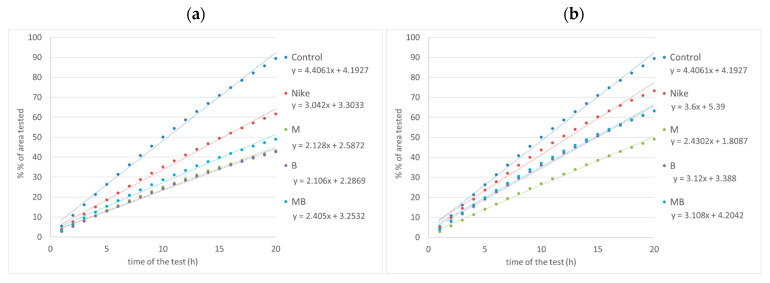
Effect on cell invasiveness of LoVo (**a**); and LoVo/DX (**b**) cancer cell lines by comparison of surface overgrowth in the evaluation of tumor cell invasiveness. Oil concentration tested 0.25 mg/mL.

The effect of flaxseed oil on the invasiveness of cancer cells of the LoVo and LoVo/DX lines was determined by comparing the surface hyperplasia in assessing the invasiveness of cancer cells. In the case of the LoVo cancer line, the lowest invasiveness of cancer cells was achieved for B and M oils. On the other hand, for the LoVo/Dx line, the lowest cell invasiveness was for M oil (Figure 9).

#### 3.2.7. Proliferation Effect of Ki67 Protein

**Figure 10:**
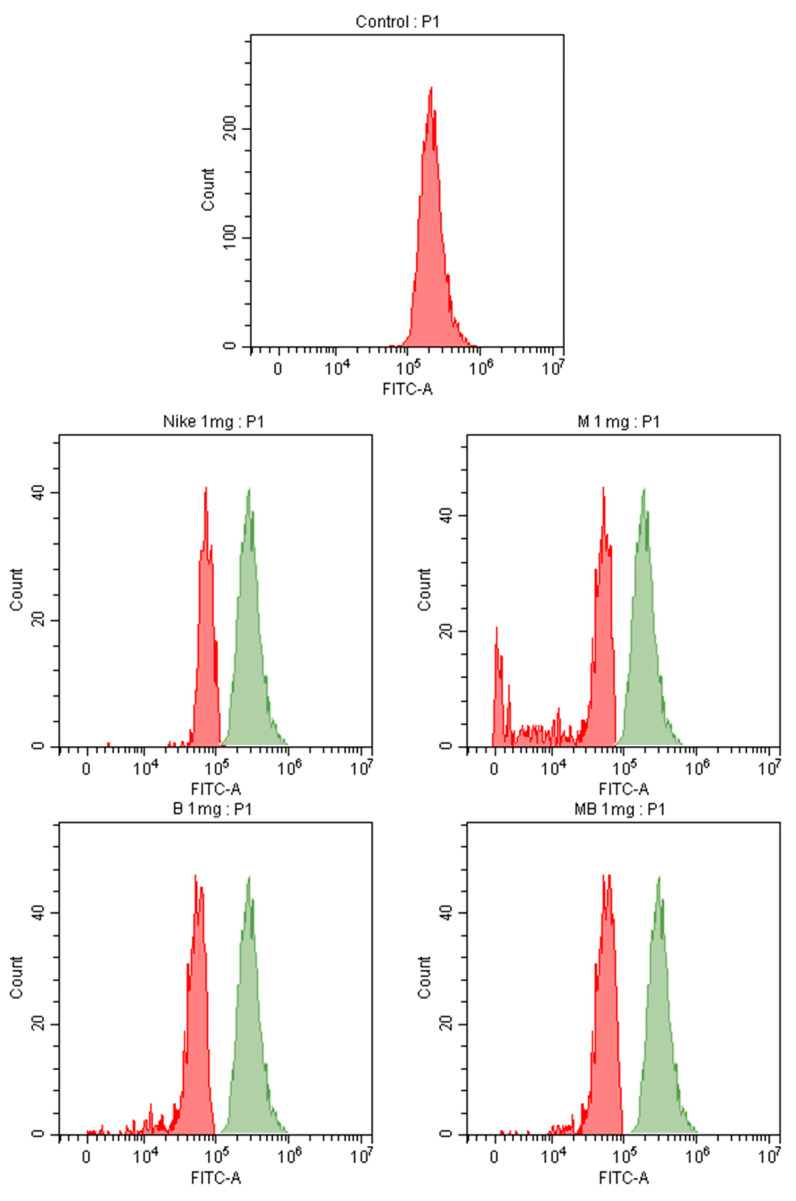
The results of the determination of the Ki67 proliferation index after treatment with the tested flaxseed oils on the LoVo cancer line cells. The red color represents the sample value, the green color represents the control value.

Ki67 is a nuclear protein associated with cell proliferation. Ki67 protein is present during all active phases of the growth cycle, but not in resting cells. Ki-67 is expressed by proliferating cells. Based on the presence of Ki67 in the cell, the percentage of circulating tumor cells in the active growth phase of the cell cycle can be estimated. Higher amounts are associated with an increased risk of progression or recurrence. The presence of Ki67 protein in tumor cells is a valuable tool to monitor the effectiveness of treatment and the risk of cancer disease progression [33,34,35]. Flow cytometric analysis revealed that the tested oils do not increase Ki67 expression, which indicates that they do not induce the metastasis of LoVo colorectal cancer cells (Figure 10).

#### 3.2.8. In Vitro Cyclooxygenase Inhibition Assay

The Cayman’s COX Colorimetric Inhibitor Screening Assay is used to evaluate the COX-1 and COX-2 inhibitory capacity of the tested oils. The results are presented as IC_50_ values, i.e., the concentration at which a 50% inhibition of enzyme activity occurred for both COX-1 and COX-2. The reference drugs were ketoprofen, ibuprofen and meloxicam. The statistical significance of COX-1 and COX-2 inhibition was calculated by a post hoc test compared to the reference drugs (** *p* < 0.01). The three drugs selected for the study were from the group of non-steroidal anti-inflammatory drugs, and inhibited COX-1 and COX-2, but to a different extent, because ketoprofen belongs to the so-called preferential COX-1 inhibitors, ibuprofen inhibits COX-1 and COX-2 to a similar extent (in the values obtained in the experiment, stronger inhibition of COX-1 is visible) and meloxicam, which is a preferential COX-2 inhibitor. The study oils showed significantly less COX-1 and COX-2 inhibition than the reference drugs. For all tested oils, stronger COX-1 inhibition was obtained compared to COX-2 inhibition. More powerful COX-1 and COX-2 inhibitory properties were obtained with oils from the transgenic lines compared to NIKE oil. The strongest COX-1 and COX-2 inhibitory properties were shown by the M oil, and lower COX-inhibiting properties, but at a similar level, were demonstrated in the B and MB oils (Table 5).

## 4. Discussion

The chemopreventive potential of flaxseed oil remains a topic of active research [38]. Research exploring the oil’s constituents and its applications suggests that a diet incorporating high concentrations of flaxseed oil can aid in cancer treatment [39]. However, when considering whole flaxseeds as a supplement, prolonged high-dose supplementation is restricted due to the presence of cyanogenic glycosides, which are toxic substances [12]. All the tested flaxseed oils, notably the M and NIKE oils, boast a significant content of PUFA, with no detectable amounts of cyanogenic glycosides. ALA is the main component of the PUFA in these oils. Originating from plants, ALA represents an n-3 fatty acid that is essential for humans as our bodies cannot synthesize it; thus, it is imperative to acquire this fatty acid from dietary sources. The ALA concentration across the evaluated oils spanned from 41.9 to 44.9% with the NIKE and M oils presenting at the higher end of this range. The health-promoting properties of ALA have been extensively documented in the scientific literature, akin to the hazards of smoking [40]. Unfortunately, the modern, highly processed diet is rich in fats of animal origin and lacks natural oils that are a source of ALA. A deficiency in essential fatty acids is the cause of disorders in bodily functions and chronic inflammation, which are often linked to civilization diseases such as cancer, type II diabetes, and cardiovascular diseases [39,41]. All the studied oils are excellent dietary sources of PUFAs, as they contain more of these acids than oils commonly used in nutrition, such as sunflower, rapeseed, rice, soybean, olive, and walnut oils [41]. The different proportions of n-6/n-3 acids in the tested oils make them suitable for various dietary and therapeutic purposes.

**Figure 11:**
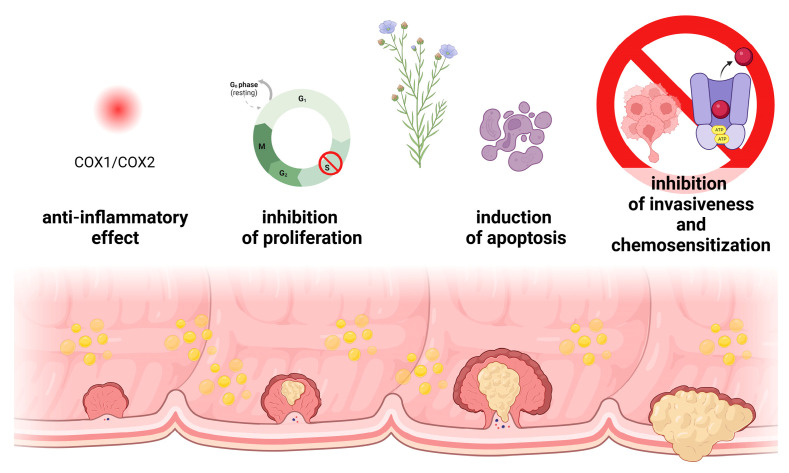
Stages of cancer progression and potential mechanisms of action of flaxseed oil. Created with BioRender.com.

ALA exerts its chemopreventive effects through various mechanisms, including proliferation, apoptosis, and migration (Figure 11) [42].

The effects of ALA include COX-dependent or COX-independent mechanisms and act on different pathways such as Wnt/β-catenin [43]. The effects of the tested oils were evident in the experiments conducted on most of the tested cell lines. All oils exhibited chemopreventive activity. The experiments also showed an effect on the apoptosis process. The effect on the induction of apoptosis in cancer cells by ALA is also described in the literature [11,42]. Many of the molecular mechanisms that increase apoptosis in cancer cells depend on the action of ALA. Primarily, ALA affects the oxidative stress state of cells. There is a relationship between anticancer activity and oxidative stress. ALA can enhance the apoptotic potential of cancer cells by increasing the concentration of intracellular reactive oxygen species (ROS), (as seen in DCFDA tests), inducing increased cell apoptosis through the loss of mitochondrial membrane potential, activation of caspase 3 and 9, and by increasing the Bax/Bcl2 ratio [3]. The observed effect is also an increase in apoptosis induced by a high concentration of the polyphenols from genetically modified flaxseeds [43].

A very important property of the tested oils is the inhibition of COX; mainly, the oils inhibited COX-1 more than COX-2. Both cyclooxygenases are biologically initiated important prostanoids, which have a potential role in the process of carcinogenesis. Significantly higher concentrations of prostaglandin E_2_ have been observed in colorectal cancer cells compared to the surrounding healthy tissue [44]. Non-steroidal anti-inflammatory drugs act as competitive COX inhibitors, blocking COX by acetylating this enzyme. The division of NSAIDs by capacity seems to be most important, in clinical terms, in inhibiting the activity of individual COX isoenzymes. NSAIDs have been shown to be of benefit in family prevention of adenomatosis, adenomas, and intestinal cancer fat [9,44]. Unfortunately, the chronic use of NSAIDs is associated, however, with an increased risk of ductal bleeding in the gastrointestinal tract and, in the case of coxibs, serious cardiovascular complications. The advantage of the tested flaxseed oils is the fact that they have COX-inhibiting properties and, at the same time, lack the side effects typical of NSAIDs. An important mechanism of action of ALA is also the modulation of COX-2 activity. Furthermore, γ-tocopherol, which is found in elevated concentrations in both the Nike and M oils, demonstrates inhibitory activity against COX-2 [45]. In in vitro model supplementation studies on Caco-2 cells, it was shown that ALA can prevent the violation of intestinal epithelial integrity by enhancing epithelial cell proliferation and reducing inflammation. As demonstrated in an epithelial wound healing assay, ALA reduced the HS-induced inflammatory response by reducing COX-2 and TGF-β mRNA expression [42]. Additionally, studies on SiHa and HeLa cervical cancer cells have shown that ALA and n-3 fatty acids regulate the growth of cervical cancer cells by reducing cell migration with a concomitant reduction in the expression of MMP-2, MMP-9, and VEGF. In addition, ALA significantly reduced the expression of NFκB and COX-2.

**Figure 12:**
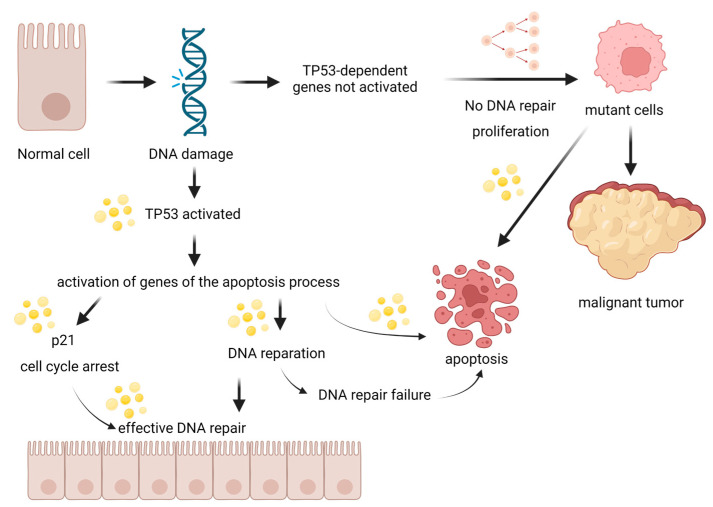
Cancer stages of apoptosis and potential sites of action of flaxseed oil. Created with BioRender.com.

Apart from apoptosis, ALA may also affect the proliferative and migratory abilities of cancer cells [12]. The strongest anti-proliferative properties were shown in the B oil for all cell lines compared to the reference drug and other oils. However, the highest values of rhodamine accumulation and the highest percentage of apoptosis for the LoVo line were observed in the M and B oils. The M oil had the highest values of p53 protein (Figure 12) and significant inhibition of COX-1 and COX-2 activity for the LoVo and LoVo/DX colorectal cancer cell lines was observed. In the M oil, there was an increased amount of phenolic substances and ALA. There is a synergistic effect between ALA and these phenolic substances, which could lead to an increase in the chemopreventive effect [12].

Other authors have also indicated the inhibition of LoVo cell growth by the linoleic acid (LA) contained in flaxseed oil in smaller amounts [46,47]. It is the second important component of the tested oil, with amounts of 19.2 to 20.9% [47].

In addition to the high content of ALA, the tested oils also contained other substances with confirmed pro-health activity. Some differences were seen between oils from transgenic and unmodified flax in the amount and composition of phytosterols, tocopherols and carotenoids. Especially, the M line oil contained significantly more polyphenols. As we have shown in our previous studies, the subtle differences in composition of the minor bioactives in oils may result in diverse effectiveness in normal and cancer cells [24,25]. The observed inhibitory impact of B oil on neoplastic cell proliferation could be partially attributed to its marginally elevated stigmasterol concentration compared to the other examined oils. Additionally, an altered fatty acid composition favoring linoleic and oleic acids in B oil, relative to Nike and M oils, may further contribute to this effect. A case–control study has indeed identified a diminished risk of ovarian cancer associated with the highest quintile intake of stigmasterol [48]. Generally, the studied flaxseed oils should be counted among the richest sources of phytosterols, and are comparable in this respect to oils such as rapeseed and sesame, and superior to soybean, sunflower, peanut, hazelnut and olive oils, tocopherols, carotenoids and polyphenols [13,14,33,49]. The latter group of compounds shows a particularly strong chemopreventive activity, protecting cells against the development of neoplastic processes and intensifying the process of apoptosis in cancer cells. The highest content of compounds from this group was found in type M plants. The compound from the group of polyphenols, which was present in the highest concentration in the tested oils, was vanillin. This compound has an antioxidant effect and a broad anti-cancer effect [50]. Importantly, vanillin exhibited anti-invasive, anti-metastatic and anti-angiogenic effects in various research models [51]. In the results derived from the assays used, M oil, characterized by a high concentration of tocopherols and polyphenolic constituents, displayed superior physicochemical properties in contrast to the other oils examined. This superior efficacy is likely due to the elevated content of phenolic derivatives present in the M oil, which demonstrates pronounced chemopreventive potential against colorectal carcinogenesis. Additionally, upon incubation with the oils, there was a discernible inhibition in cellular motility. The most pronounced inhibitory effect was observed with oil derived from type M plants, which have the highest vanillin concentration. Flaxseed oils demonstrate both anti-inflammatory and anti-neoplastic properties, targeting the hallmark traits of neoplastic cells, such as cellular proliferation, apoptosis induction, and migratory capacity. The bioactivity of the predominant constituents of flaxseed oil, as elucidated above, is corroborated by the published literature discussing its antineoplastic potential [38,52].

Several research groups have elucidated the chemopreventive potential of various plant oils. Notably, extensive research underscores the profound chemopreventive efficacy of olive oil, which has demonstrated inhibitory activities across the initiation, progression, and carcinogenesis phases [6]. The chemopreventive aptitude of olive oil is attributed to its robust antioxidant prowess, coupled with its capacity to deter proliferation and induce apoptosis across diverse oncogenic cell lines. These effects have been corroborated in leukemia, breast, and colorectal malignancies. What is more, olive oil may play a role in epigenetic therapy by altering NF-κB and apoptotic pathways via targeting the noncoding RNAs and methylation machinery that affect epigenome to prevent colon carcinogenesis [53]. Olive oil is poor in ALA, although, similar to flaxseed oil, contains oleic and linoleic acids, which may have cancer-suppressing potential. Their consumption caused the induction of apoptosis and cell differentiation mediated by early COX-2 downregulation followed by a reduction in Bcl-2 expression [54]. A significant inverse correlation was found between the risk of colon cancer and higher plasma concentrations of oleic and linoleic acids and ALA in a Singaporean Chinese Health Study.

This study has several limitations. Primarily, it was confined to in vitro assessments to gauge the chemopreventive effects concerning colorectal cancer. From our in vitro results, it is inferred that significant doses of modified flaxseed oil must be consumed over extended periods to achieve the desired chemotherapeutic effect in preventing colorectal cancer. However a dose that may seem excessive is already recommended in the Cancer Prevention Diet, also known as the Bill Henderson Protocol (OHS), and consists of eating raw food daily in combination with cottage cheese and flaxseed oil (six tablespoons of flaxseed oil) [55]. The effects of drinking flaxseed oil have been confirmed in many clinical studies. Depending on the goal to be achieved, such as lowering blood pressure, improving the lipid profile [56] or lowering the inflammation (C-reactive protein), there are different recommendations for the dose of unmodified flaxseed oil and the time of use. For example, in patients diagnosed with mild hypercholesterolemia who were on a lipid-lowering diet with the addition of flaxseed oil at a dose of 15 mL daily for four weeks, significantly decreased low-density lipoprotein (LDL) levels and increased HDL cholesterol [57] were observed.

However, we emphasize that this research merely serves as a precursor to forthcoming preclinical studies. Although the flaxseed oil doses appear excessive, their exact amounts will undergo validation in subsequent in vivo experiments, followed by clinical trials targeting the tumor directly. In our current in vitro model, we utilized a 2D paradigm, which limits our interpretations to a direct tumor effect.

The ultimate goal is to harness these oils as adjuncts in colorectal cancer therapy, specifically as colon-targeted formulations. In the large intestine, it is anticipated that substantial amounts of oil will be liberated. This is particularly relevant given the propensity of cancer cells to assimilate oils, subsequently influencing their metabolic pathways.

## 5. Conclusions

The data generated from our investigations strongly suggest that oil derived from type M plants holds the potential for incorporation into chemopreventive formulations. The empirical evidence underpins the hypothesis that the dietary integration of flaxseed oil can offer a protective mechanism against the initiation of carcinogenic pathways within the colonic cellular architecture. This protection is postulated to be mediated through the induction of apoptotic processes. In scenarios of advanced neoplastic stages, flaxseed oil exhibits suppressive capacities—demonstrating late-stage chemoprevention—by mitigating the motility of malignant cells. Furthermore, owing to its enriched polyphenolic profile, flaxseed oil from the M genotype not only presents potential oncologic protective effects but also may serve as a countermeasure against the initiation of inflammatory and autoimmune-mediated enteropathies.

## Figures and Tables

**Table 1:** Fatty acids composition and phytosterol content in flaxseed oils: Nike, B and M.

Oil	NIKE	B	M
Fatty acids [%]			
C16:0	5.54 ± 0.11	5.45 ± 0.18	5.45 ± 0.16
C18:0	6.12 ± 0.05	6.06 ± 0.06	5.96 ± 0.06
C18:1 n-9	23.20 ± 0.10	24.60 ± 0.10	23.60 ± 0.10
C18:2 n-6	19.20 ± 0.10	20.90 ± 0.20	19.70 ± 0.30
C18:3 n-3	44.90 ± 0.50	41.90 ± 0.30	44.40 ± 0.50
Others ∑	1.00 ± 0.00	1.10 ± 0.00	0.90 ± 0.00
SFA	12.3	12.2	12
MUFA	23.5	24.8	23.9
PUFA	64.1	62.9	64.1
n-6/n-3	0.431	0.499	0.444
Phytosterols [mg/100 g]			
Campesterol	128.8 ± 2.8	121.5 ± 5.9	121.1 ± 4.3
Stigmasterol	40.2 ± 0.7 ^a^	42.4 ± 1.3 ^b^	27.4 ± 0.5 ^ab^
Obustifoliol	19.8 ± 0.3	17.2 ± 0.2	16.6 ± 0.5
β-Sitosterol	291.9 ± 4.9 ^a^	280.2 ± 13.5 ^ab^	296.6 ± 7.5 ^b^
Δ5-Avenasterol	56.4 ± 2.8	51.2 ± 0.9	49.8 ± 1.9
Cycloartenol	88.0 ± 1.7	86.2 ± 3.5	81.7 ± 2.1
24-Methylenecycloartanol	56.6 ± 1.2	56.1 ± 0.7	58.7 ± 1.4
Total	681.7 ± 11.7 ^ab^	654.8 ± 25.5 ^a^	652.0 ± 17.3 ^b^

The statistical analysis was performed using the one-way ANOVA test, differences between oils were considered statistically significant at ^a^: *p* < 0.05 and with ^b^ at *p* < 0.005. SFA—saturated fatty acids, MUFA—monounsaturated fatty acids, PUFA—polyunsaturated fatty acids.

**Table 2:** The antioxidant composition and content in flaxseed oil Nike, B and M.

Antioxidant Composition	NIKE	B	M
Tocopherols [mg/kg]			
α-Tocopherol	13.61 ± 0.58	9.60 ± 1.58	11.93 ± 0.76
γ-Tocopherol	330.86 ± 3.19	313.32 ± 18.99	327.26 ± 21.40
δ-Tocopherol	10.20 ± 0.28	11.18 ± 1.01	11.08 ± 0.74
Total	354.67 ± 3.75 ^a^	334.11 ± 20.90 ^a^	350.27 ± 21.46
Plastochromanol-8 [mg/kg]	107.71 ± 7.84 ^b^	89.65 ± 9.79	80.50 ± 15.12 ^b^
Carotenoids [mg/kg]			
all-trans-β-Carotene	4.51 ± 0.58	4.05 ± 0.34	2.05 ± 0.18
all-trans-Lutein + all-trans-Zeaxanthin	18.82 ± 0.73	17.31 ± 1.59	19.18 ± 0.36
all-trans-Neoxanthin	0.57 ± 0.06	0.51 ± 0.01	0.56 ± 0.08
all-trans-β-Cryptoxanthin	0.19 ± 0.01	0.18 ± 0.01	0.20 ± 0.00
Total	25.03 ± 0.24	22.86 ± 1.94	22.95 ± 0.29
Polyphenols [µg/kg]			
Vanillic acid	32.78 ± 1.78 ^a^	45.91 ± 1.17	58.68 ± 5.00 ^a^
Vanillin	95.40 ± 15.45 ^b^	95.67 ± 17.24 ^c^	145.91 ± 26.94 ^bc^
p-Coumaric acid	20.98 ± 0.37	5.72 ±0.37	5.16 ± 0.54
Syringaldehyde	6.23 ± 1.07	12.47 ± 1.70	16.07 ± 0.55
Ferulic acid	48.66 ± 3.02 ^a^	28.17 ± 3.50 ^a^	30.42 ± 2.17
Coniferyl aldehyde	23.35 ± 2.40	32.60 ± 3.32	37.47 ± 2.73
o-Coumaric acid	3.47 ± 0.49	6.13 ± 0.34	6.99 ± 0.29
SECO *	7.47 ± 0.74	7.91 ± 1.57	8.06 ± 1.50
Total	238.34 ± 19.84 ^b^	234.57 ± 26.08 ^c^	308.77 ± 34.79 ^bc^

The statistical analysis was performed using the one-way ANOVA test, differences between oils were considered statistically significant at ^a^: *p* < 0.05, ^b,c^: *p* < 0.0001. * SECO—secoisolariciresinol.

**Table 3:** Cytotoxic effect after the incubation of cells with the tested linseed oils and reference drug.

Cell Line	IC_50_ [mg/mL]				IC_50_ [μg/mL]
	Nike	M	B	MB	Doxorubicin
CCD 841 CoTr	NA	NA	NA	NA	33.2 ± 2.10
LoVo	4.30 ± 0.60	1.54 ± 0.51	1.58 ± 0.40	1.54 ± 0.47	1.7 ± 0.42
LoVo Dx	6.21 ± 2.90	1.58 ± 0.39	2.80 ± 0.61	3.49 ± 0.67	32.04 ± 3.5
A549	4.34 ± 1.27	NA	3.03 ± 0.95	NA	3.94 ± 0.7
MCF7	1.28 ± 0.21	1.62 ± 0.37	0.71 ± 0.05	1.17 ± 0.20	2.88 ± 0.46
CCRF/CEM	0.67 ± 0.02	0.28 ± 0.01	0.38 ± 0.06	0.80 ± 0.03	1.48 ± 0.22

**Table 4:** Correlations between inhibitory concentrations for cell lines and the content of individual compounds from the polyphenol group (* *p* < 0.05).

	Polyphenols							
	Total	Vanilic Acid	Vanilin	p-Coumaric Acid	Syringaldehyde	Ferulic Acid	Coniferyl Aldehyde	o-Coumaric Acid
LoVo	−0.50 *	−0.75 *	−0.56 *	0.98 *	−0.84 *	0.73 *	−0.74 *	−0.81 *
LoVo Dx	−0.11	−0.43 *	−0.18	0.95	−0.55	0.91 *	−0.42 *	−0.53 *
MCF7	−0.48 *	−0.39	−0.63 *	0.18	−0.36	0.02	−0.27	−0.26
CCRF/CEM	0.57 *	0.31	0.44	0.53	0.17	0.90 *	0.35	0.24

**Table 5:** COX inhibition was calculated for COX-1 and COX-2 enzymes after incubation with the tested oils and reference drugs.

	IC_50_ [µg] (SD)		COX-2/COX-1 Selectivity Ratio
	COX-1	COX-2	
Nike	1381.89 ± 7.83	2838.07 ± 709.11	1.92
M	944.1 ± 61.31	1813.96 ± 22.35	1.68
B	1307.20 ± 159.20	2315.40 ± 180.09	1.72
MB	1338.93 ± 103.34	2243.73 ± 378.89	2.12
Meloxicam	116.89 ± 5.62	103.28 ± 8.30	0.88
Ibuprofen	111.18 ± 2.35	149.34 ± 11.32	1.34
Ketoprofen	74.39 ± 1.20	92.29 ± 5.89	1.24

## Data Availability

Data availability statements are available after contact with corresponding author: tomasz.gebarowski@upwr.edu.pl.
